# Supplementary material for: Causes of and Alternatives to Medication for Behaviours That Challenge in People with Intellectual Disabilities: Direct Care Providers’ Perspectives
Source: Int J Environ Res Public Health. 2022 Aug 13;19(16):9988. doi: 10.3390/ijerph19169988 (PMC9408416; doi:10.3390/ijerph19169988)
Supplement: Supplementary file 1 [file ijerph-19-09988-s001.zip › ijerph-1800601-supplementary.pdf]

Topic Guide for SPECTROM focus groups 1 & 2

Aims

To explore views relating to problem behaviour
Attribution issues (locus of control, beliefs about controllability)
Triggers
Understanding client's inner world-how do they see the world
Sensory issues
Communication issues (including false +vs)
Care staff responses (including emotional) to problem behaviour

Psychiatric issues
Difference between PI and behaviour problems,
PI causing behaviour problem
PI misinterpreted as behaviour problem
Behaviour problem misinterpreted as a PI

Medication
Understanding of indication for drug use
Pros and cons of long term use of drugs
Views on adverse effects
Views & anxieties around withdrawal
What is care staff's view on their influence on prescribing (locus of control as for prescribing)?
Process of seeking/maintaining/accessing medication prescription

Small case vignettes

A 19 years old man with moderate ID and ASD became aggressive towards his parents who asked the doctor for a prescription. However, further assessment revealed this young man's obsession with water and fear of noise.
[This is an example of contextual factors affecting behaviour, which does not need medication.]

A 32 years old man with moderate ID who does not speak, was brought to the psychiatric clinic. He was on multiple psychotropic medications for several years including a number of antipsychotics at a high dose along with a regular dose of procyclidine. Examination revealed that the man had severe tremor and dyskinetic body movement including movements in facial muscles.
[This is an example of the downside of using multiple psychotropics for many years.]

A 33 years old man with moderate ID but no speech showed challenging behaviour periodically and the carers raised the issue whether this is part of a bipolar disorder. However, the clinician was not certain as there were no other symptoms to support the diagnosis of bipolar disorder apart from the periodic nature of the behaviour.
[This raises the issue of the confusion between challenging behaviour & a psychiatric illness.]

Topic Guide

Aim 1

Opening question?

To what extent is problem behaviour part of your working life?

- What does this behaviour look like?
  [Agree on a definition of problem behaviour/a frame of reference]
- Who exhibits this behaviour?
- How often does it occur?

What do you think triggers/causes this behaviour?

- To what extent do you think that problem behaviour is communicative/the client is trying to tell you something?
- To what extent is problem behaviour part of learning disabilities?
  [Explore internal and external triggers]

To what extent do you think that the person is in control of their own behaviour?

- Why do they act aggressively towards you/others?
  [Use specific behaviours to probe understanding of client's inner world]

What do you think helps with problem behaviour?

- What do you think *could* help with problem behaviour?

How do you help a client who is showing problem behaviour?

- What do you do immediately/during?
- What do you do afterwards?
- What do you do if the behaviour continues?
  [Explore actions taken both during and after the behaviour]

To what extent do you think you can help/intervene with behaviour?

- How effective do you think your actions are?
- Do you think what you do helps?

How do you feel when a client shows problem behaviour?

- During?
- Afterwards?
- How does it make you feel about your job?
  [Don't spend too much to time on this – well covered in the literature]

(Remember to check often with the group that they share that experience.)

Aim 2

To what extent do you think problem behaviour is related to psychiatric diagnoses?

- Can you give me some examples?
  [Check which diagnoses participants are referring to – e.g. ASD, psychosis, depression etc rather than learning disability]
  Do you think any of your clients have mental illness (that is not diagnosed)?
- Do mental health problems cause problem behaviour in people with learning disabilities? To what extent?

Do you think problem behaviour is a psychiatric illness?

Do you think that all problem behaviour is caused by a psychiatric illness?

Aim 3

Are any of your clients taking medication for problem behaviour?

- Can you tell me more?
- Which medications are they taking?
  [Establish frame of reference for psychotropic medication]
- For which types of behaviour?

Do you know the reasons why your clients take medication for behaviour?

- Is the indication clear?
- To what extent are these medications for mental illness?

How did your clients start taking medication for their behaviour?

- Can you describe the process?
- Who first sought help?
- Why?
- Who did your client/you see?
- Who prescribed the medication?

Whose responsibility do you think is to decide about the use of psychotropic medication?
[clarify what is meant by psychotropic, if required]

Could you play any role in the process of prescribing?

- If so, how?

Who decides whether your clients should carry on taking medication?

- Can you describe the process?
- What is your role in this?

What do you think about people with learning disabilities taking medication for problem behaviour?

- Do you think it works? If so, what for? If not, why not?
- Are there any problems associated with taking these types of medication?
- Are you aware of side effects? How much so they affect people with learning disabilities?

How do you think people with learning disabilities feel about taking medication for problem behaviour?

People with learning disabilities often take medication for problem behaviour for a long time, what do you think about this?

- Are you aware of side effects?

What are the alternatives to taking medication?

- Do you think they work? If so, what for? If not, why not?

How would you feel about withdrawing medication that is prescribed for problem behaviour?

What happens when a client stops taking medication that is prescribed for problem behaviour?

- What would be the process – can you describe it?
- Who would be involved?
- Who’s decision would it be? Who asks for it?
- What do you think would happen to the client?

- How would you feel?
- What support would the client need?
- What support would you need?

Do you know of any clients who have come off/reduced medication that was prescribed for problem behaviour?

- What happened?
  [Explore positive and negative experiences]
- Who was involved
- Who made the decisions/took the action?

Finally, we have covered a lot of ground today but are there any other issues or points you would like to raise that we have not discussed?
